# The Spectrum of MRI Findings in Dengue Encephalitis

**DOI:** 10.7759/cureus.29048

**Published:** 2022-09-11

**Authors:** Priyal LNU, Vineet Sehgal, Lucky Bhalla Sehgal, Nihal Gulati, Saniya Kapila

**Affiliations:** 1 Neurology, Sehgal's Neuro & Child Care Centre, Amritsar, IND; 2 Neurology, Lady Hardinge Medical College, Delhi, IND; 3 Neurology, Amandeep Medicity, Amritsar, IND; 4 Paediatrics, Sehgal's Neuro & Child Care Centre, Amritsar, IND; 5 General Practice, Navpreet Hospital, Amritsar, IND; 6 General Practice, Fortis Escorts Hospital, Amritsar, IND

**Keywords:** expanded dengue syndrome, infectious encephalitis, mri findings, 3-tesla mri, dengue fever/complications

## Abstract

Background

In this study, we aimed to describe eight cases of dengue encephalitis along with their magnetic resonance imaging (MRI) findings. Dengue encephalitis is caused by an arbovirus that has four strains DENV1-DENV4. The dengue virus is usually non-neurotropic but DENV2 & DENV3 are neurotropic. Dengue encephalitis is characterized by headaches, seizures, and altered consciousness.

Methodology

At our facility, we performed 3T MRI on eight suspected cases of dengue encephalitis using the criteria established by Varatharaj et al. We were able to diagnose dengue encephalitis based on the proposed criteria which included symptoms, serology, cerebrospinal fluid (CSF) analysis results, MRI findings, and routine blood laboratory workup in dengue encephalitis. Because numerous brain regions are potentially impacted in severe cases of dengue encephalitis, an MRI of the brain can reveal the severity of the condition. In deteriorating situations, it may detect whether or not further regions are being impacted. Hence, MRI should be done in all suspected cases of dengue encephalitis.

Results

The changes observed on MRI of the eight cases were in the supra-tentorium (deep periventricular white matter, subcortical white matter, and deep gray matter of the brain, which includes basal ganglia and thalami), infra-tentorium (cerebellar white matter and brainstem, which includes pons), and occasionally in cortical gray matter. The MRI showed mild-to-moderate hyperintensities on T2-weighted images and fluid-attenuated inversion recovery sequence (FLAIR); diffusion restriction is seen on diffusion-weighted images. The neurological clinical features included non-localizing signs and symptoms such as altered mental status, headache with vomiting, and fever.

Conclusions

The commonly affected areas of the brain in dengue encephalitis are the basal ganglia, thalamus, brainstem, cerebellum, cortical white matter, periventricular white matter, and cortical gray matter, which are all hyperintense on T2-weighted images and FLAIR. The lesions are iso or hypointense on T1-weighted images and micro-hemorrhages appear as blooming on susceptibility-weighted MRI. MRI is a crucial initial investigation in suspected cases of dengue encephalitis and known cases of dengue fever experiencing worsening neurological conditions.

## Introduction

Dengue fever is a mosquito-borne (*Aedes aegypti*) disease caused by an arbovirus. On a spectrum, the mildest disease is a fever and rash (also called breakbone fever), and the most severe form is dengue shock syndrome with multisystem dysfunction. It is seen in tropical and subtropical areas [1]. There are four strains of dengue virus, namely, DENV1, DENV2, DENV3, and DENV4. Dengue virus is not commonly known to be neuro-virulent. However, the DENV2 and DENV3 strains of the dengue virus are neurotropic [2].

Dengue fever outbreaks occur in India in the rainy season from July through November. The participants included in our study reflect the whole experience of our team of healthcare providers from April 2022 to May 2022. India is categorized as the endemic category A for dengue fever by the World Health Organization (WHO). This year, from April through May 2022, we saw a spike in the number of dengue cases presenting with neurological complaints to our facility. Some neurological manifestations of dengue fever are Guillain-Barré syndrome, dengue encephalitis, dengue encephalopathy, stroke, neuro-ocular manifestations (such as maculopathy, papilledema, ocular paresis), transverse myelitis, acute disseminated encephalomyelitis (ADEM), cerebral edema, and cerebral herniation [3]. When compared to dengue encephalopathy, dengue encephalitis is rarer. The overall incidence of dengue encephalitis is 6.2%. Dengue encephalitis is characterized by headaches, seizures, and altered consciousness.

## Materials and methods

All patients presenting to us with similar neurological complaints and concomitant dengue fever have been reported in this article. In eight patients with dengue encephalitis between the ages of seven through 40 years (five males and three females), we looked at their 3T magnetic resonance imaging (MRI) films and the various sequences (T1, T2, T1 with contrast, fluid-attenuated inversion recovery sequence (FLAIR), diffusion-weighted images (DWI) and susceptibility-weighted images (SWI)). The most important clinical and lab features observed in these patients were fever, altered mental status, a positive serum enzyme-linked immunosorbent assay (ELISA) for dengue, and cerebrospinal fluid (CSF) lymphocytic pleocytosis, with normal glucose and slightly elevated proteins.

We used the case definition of dengue encephalitis as proposed by Varatharaj et al. to diagnose our patients [4]. According to the case definition, dengue encephalitis can be diagnosed by the presence of evidence of dengue virus in the body or brain which is done by a positive dengue virus culture and/or dengue immunoglobulin M (IgM) antibodies in either serum or CSF (or both), or the presence of neuroimaging evidence suggestive of dengue encephalitis.

Based on the literature review, the typical areas involved in the brain are the ganglia-thalamic complex, cerebellum, and cortical gray and white matter. The lesions are hyperintense on T2-weighted images and FLAIR images, and hypo or isointense on T1-weighted images.

In addition to this, Varatharaj’s case definition of dengue encephalitis also includes the presence of clinical and laboratory features such as fever, headache, and reduced level of consciousness that are not explained by acute liver failure, shock, electrolyte derangements, and micro or macro-intracranial hemorrhages.

## Results

**Figure 1:**
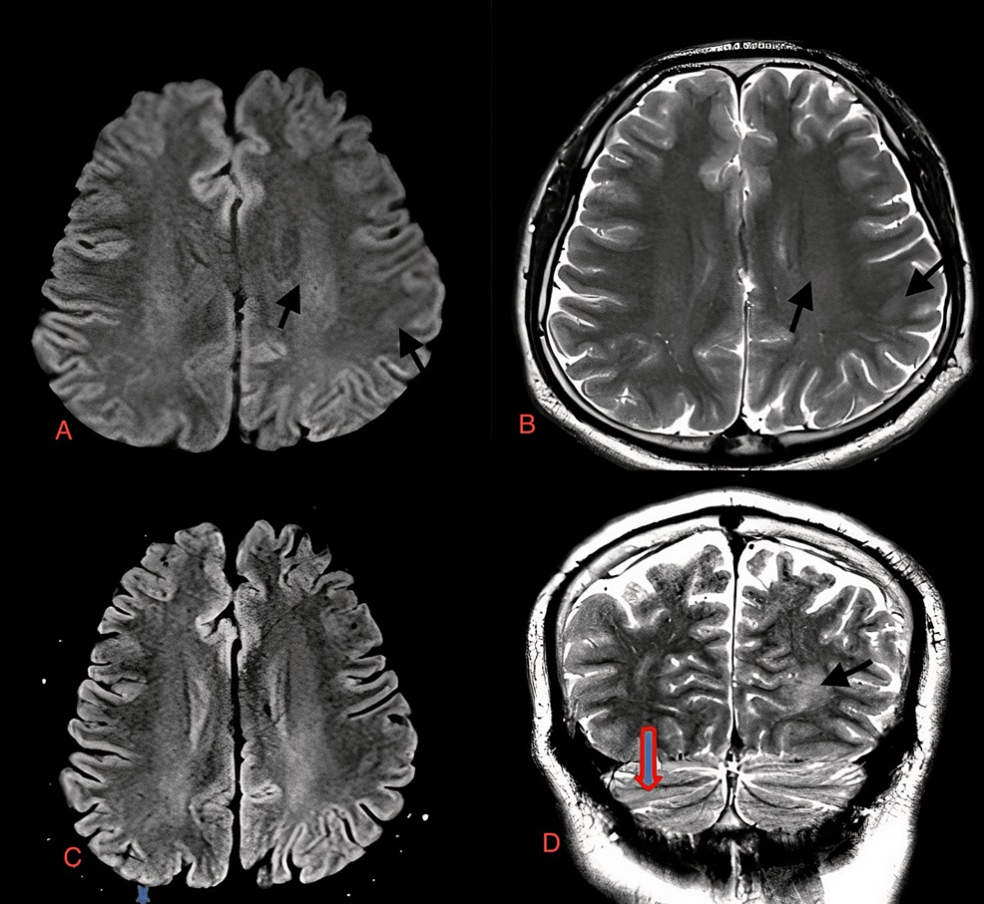
Case 2: Asymmetrical hyperintensities are seen in the deep and subcortical white matter of the left parietal lobe on DWI axial (A) and T2W axial (B) images in a 28-year-old male with dengue encephalitis. An area of cerebellar hyperintensity is present in the T2W image (D). No diffusion restriction is seen in the FLAIR image (C). T2W: T2-weighted image; DWI: diffusion-weighted image; FLAIR: fluid-attenuated inversion recovery sequence

**Figure 2:**
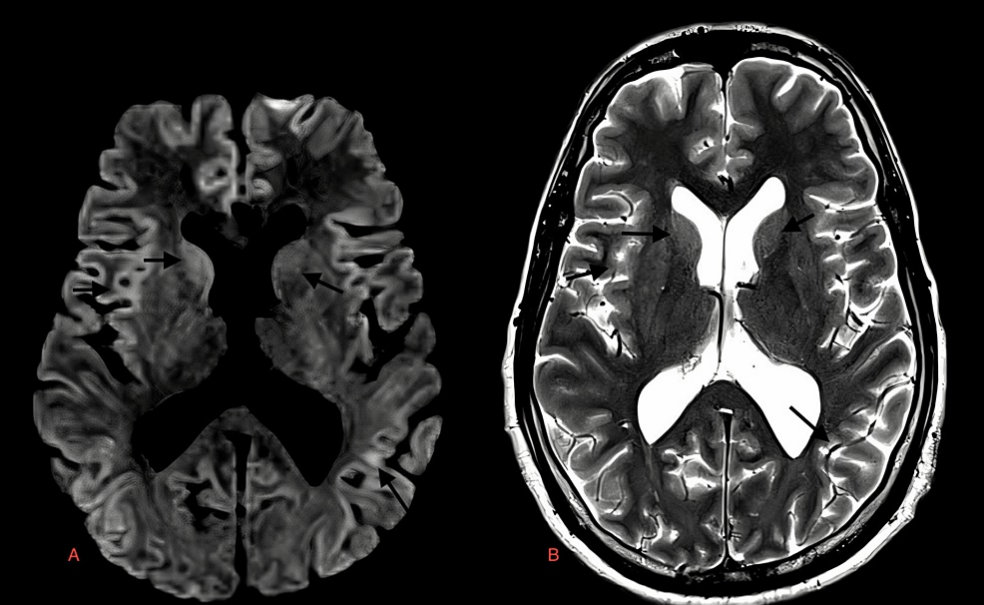
Case 3: DWI and T2W images with diffusion restriction and axial hyperintensities, respectively, in both cerebral hemispheres and both caudate nuclei (black arrows in A & B, respectively), MRI of a 36-year-old male patient with dengue encephalitis. DWI: diffusion-weighted image; T2W: T2-weighted image; MRI: magnetic resonance imaging

**Figure 3:**
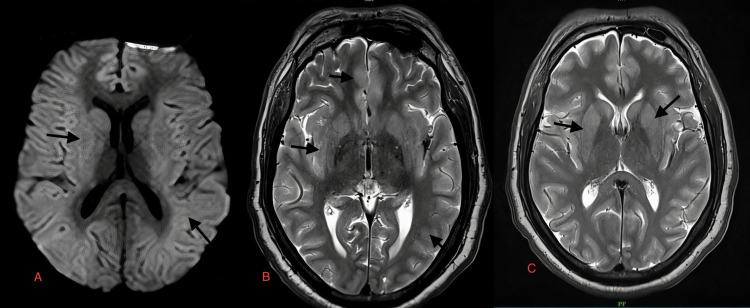
Case 4: Diffuse areas of diffusion restriction in DWI image are seen in both cerebral hemispheres including gray white matter, deep white matter, and basal ganglia region with sparing of the thalami and frontal white matter (A) in a 17-year-old male with dengue encephalitis. Corresponding hyperintensities are seen on T2W images (black arrows in B and C). DWI: diffusion-weighted image; T2W: T2-weighted image

**Figure 4:**
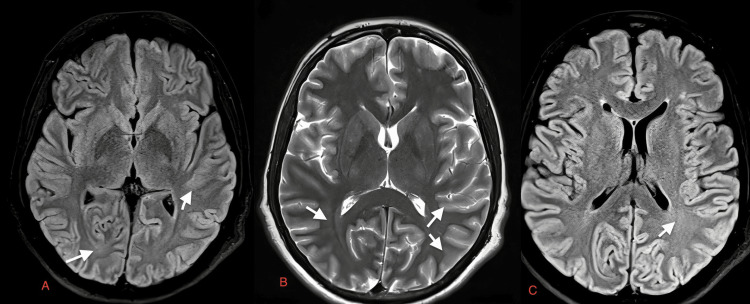
Case 5: Cortical areas of hyperintensities are observed in both parieto-occipital lobe on T2W (white arrows in B) and FLAIR images (white arrows in A and C) of a 40-year-old male patient on day one of presentation to the hospital. T2W: T2-weighted image; FLAIR: fluid-attenuated inversion recovery sequence

**Figure 5:**
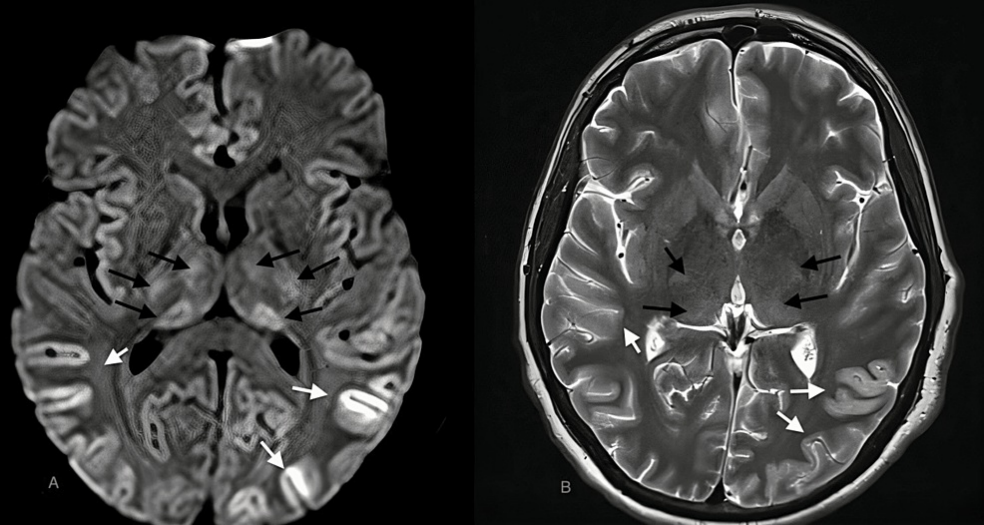
Case 5: New interval increase in hyperintense areas in DWI and T2W images are identified in both parieto-occipital lobes and thalami of a 40-year-old male patient six days after presentation with dengue encephalitis (white arrows and black arrows, in A and B, respectively). DWI: diffusion-weighted image; T2W: T2-weighted image

**Figure 6:**
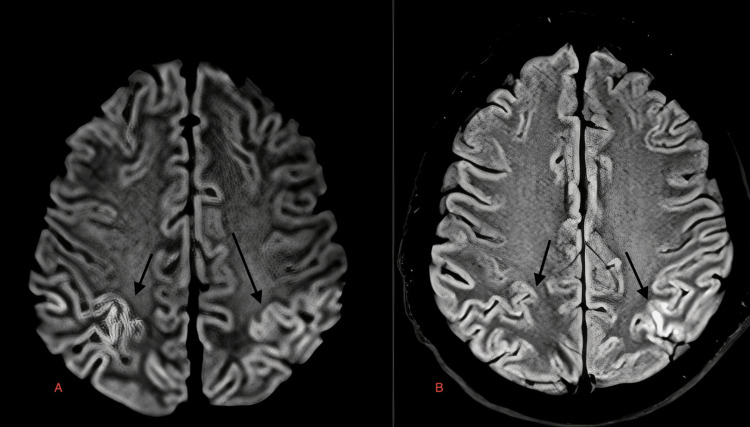
Case 5: Interval increase in the cortical hyperintensities are seen in both parieto-occipital lobe on DWI and FLAIR images (black arrows in A and B, respectively) of a 40-year-old male patient six days after presentation with dengue encephalitis. DWI: diffusion-weighted image; FLAIR: fluid-attenuated inversion recovery sequence

**Figure 7:**
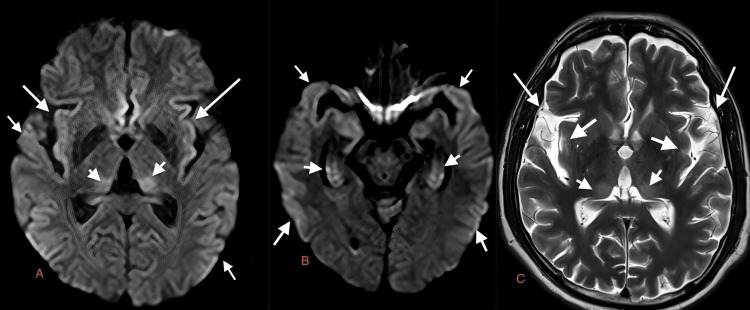
Case 6: Diffuse gyral areas of diffusion restriction are seen in both cerebral hemispheres on DWI (white arrows in A and B), predominantly in the temporal lobe also involving both hippocampi on day one of presentation in a 15-year-old male patient with dengue encephalitis. Areas of diffusion restriction are also seen in both thalami on DWI with corresponding hyperintensities on T2W images (white short arrows in A, B, and C, respectively). T2W: T2-weighted image; DWI: diffusion-weighted image

**Figure 8:**
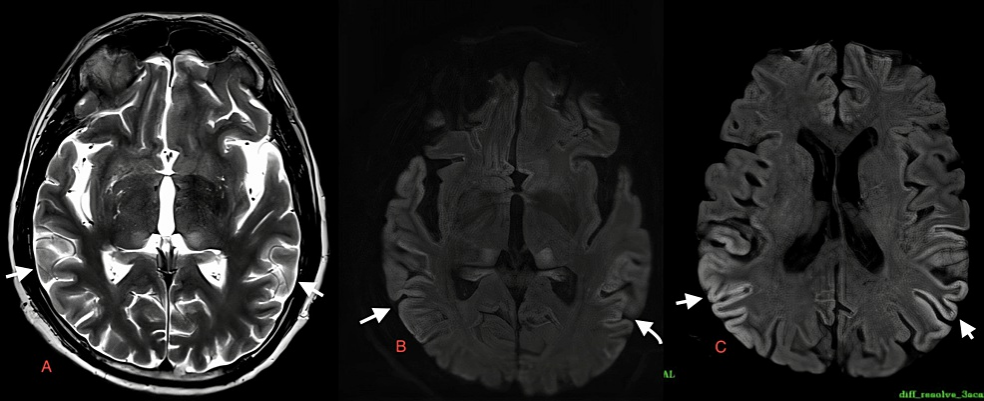
Case 6: MRI of a 15-year-old male patient on day six of admission. Increased gyral hyperintensities are observed in the posterior parietal lobe on T2W and DWI (white arrows in A, B, C). T2W: T2-weighted image; DWI: diffusion-weighted image; MRI: magnetic resonance imaging

**Figure 9:**
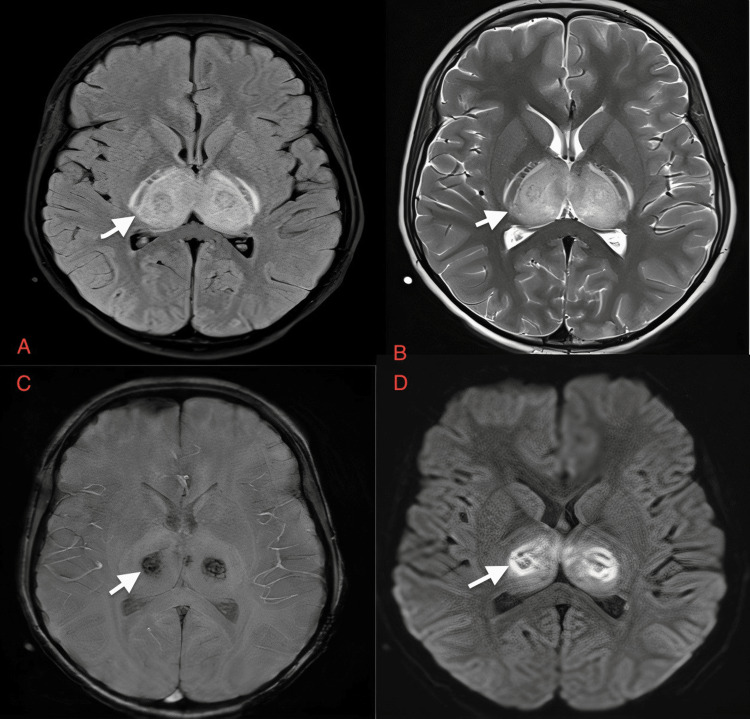
Case 8: Symmetrical hyperintensities are seen on FLAIR and T2W (white arrows in A and B, respectively) images in both thalami in a nine-year-old male patient on day one of presentation with dengue encephalitis. Central areas of blooming indicating hemorrhage are seen on SWI (C). Diffusion restriction is also seen on DWI (white arrow in D). FLAIR: fluid-attenuated inversion recovery sequence; T2W: T2-weighted image; SWI: susceptibility-weighted image; DWI: diffusion-weighted image

**Figure 10:**
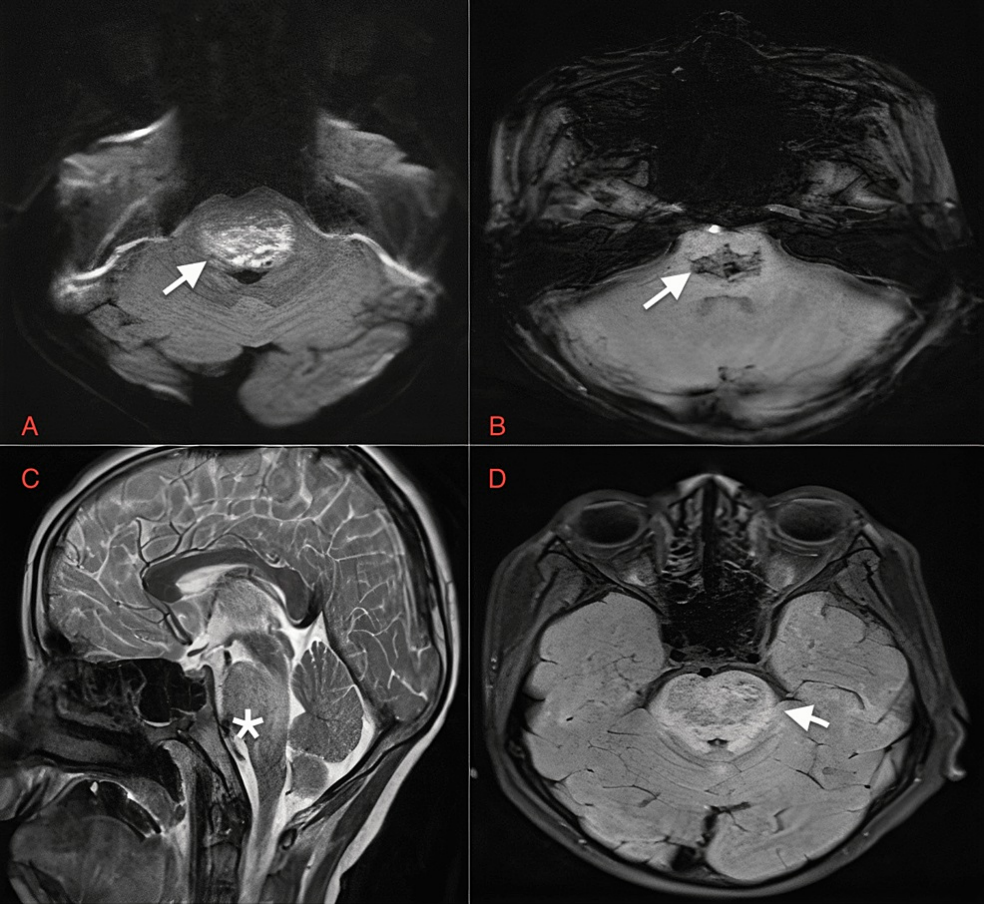
Case 8: Diffusion restriction is also seen on DWI in the pons (white arrows in A). Areas of blooming are seen on SWI (white arrow in B). Hyperintense areas are seen in pons T2W sagittal images and FLAIR images (white arrow in C and D) in a nine-year-old male patient with dengue encephalitis. DWI: diffusion-weighted image; SWI: susceptibility-weighted image; T2W: T2-weighted image; FLAIR: fluid-attenuated inversion recovery sequence

Eight patients presented to us with dengue fever (syndrome or fever, retro-orbital pain, body aches, joint pains, and the presence of thrombocytopenia) with severe neurological features such as altered mental status, seizures, and headache with vomiting. The sample consists of male and female patients between seven to 40 years of age. There was no significant medical, family, or psychosocial history. The patients’ medical histories did not include significant head injuries or past neurosurgical interventions. The signs and symptoms of the illness began with a fever and generalized body aches and then progressed to altered mental status or seizures within three to four days. The serum electrolytes, liver function tests, kidney functions, complete blood count with differential cell counts and peripheral blood smear, basic metabolic panel, C-reactive protein, prothrombin time, international normalized ratio, CSF lab tests, cell counts, proteins, sugar, adenosine deaminase, cytology, and MRI were analyzed as part of the management of each case. By performing blood cultures 1 and 2, IgM ELISA for leptospirosis, Widal test, and pan-neurotropic virus panel (pan-flavivirus RNA, pan-herpes DNA, pan-paramyxovirus RNA, human parvovirus B19, human adenoviruses, human para-echoviruses), all causes of fever with thrombocytopenia were ruled out (Figures 1-10).

Dengue infection was ascertained with serum and CSF dengue serology (IgM ELISA). A card test was not done due to its high false positivity. Even so, other viral infections such as Japanese encephalitis (JE), herpes simplex encephalitis, enteric fever, and scrub typhus were ruled out with the help of ELISA, and exposure to dengue was confirmed.

If the patient experienced seizures, they were provided with supportive care in the form of intravenous fluids, antipyretics, and antiepileptics. After developing the input and output chart, we continued to keep a close eye on all of the blood tests until the signs and symptoms improved. Improvements, as determined by the neurologist and patient-reported outcomes, were used to guide subsequent treatment, discharge planning, and follow-up. Concerning this case series, we got written permission from the patients for sharing the details of their cases and MRI films in our article. We made sure their identities are not disclosed in the report (Table 1).

**Table 1:** Eight cases of dengue encephalitis with neurological signs and symptoms and corresponding MRI findings. MRI: magnetic resonance imaging; IgM: immunoglobulin M; SWI: susceptibility weighted imaging

Ages and sex	Symptoms and signs	Serology and CSF status	MRI findings
A 12-year-old female	Fever, retro-orbital pain, seizures	Serum positive for dengue, CSF negative for dengue IgM, slightly elevated CSF protein and cell, and normal glucose	Within normal limits
A 28-year-old male	Fever, body ache, altered sensorium	Serum and CSF positive for dengue IgM	Asymmetrical hyperintensities are seen in deep and subcortical white matter of left parietal lobe on FLAIR axial (Figure 1A) and T2W coronal (Figure 1B) in a 28-year-old male with dengue encephalitis. An area of cerebellar hyperintensity is present in the T2W image (Figure 1D). No diffusion restriction is seen in the FLAIR image (Figure 1C).
A 36-year-old female	Fever, altered mental status	Serum positive for dengue IgM, CSF lymphocytic pleocytosis, and normal glucose	Diffuse areas of diffusion restriction in FLAIR image are seen in both cerebral hemispheres including gray-white matter, deep white matter, and basal ganglia region with sparing of thalami and frontal white matter (Figure 2A) in a 17-year-old male with dengue encephalitis. Corresponding hyperintensities are seen on T2W images (black arrows in Figure 2B and Figure 2C)
A 17-year-old female	Fever, vomiting, headache	Serum positive for dengue IgM, CSF lymphocytic pleocytosis, normal glucose, and slightly elevated proteins	Diffuse areas of diffusion restriction in FLAIR image are seen in both cerebral hemispheres including gray-white matter, deep white matter, and basal ganglia region with sparing of thalami and frontal white matter (Figure 3A) in a 17-year-old male with dengue encephalitis. Corresponding hyperintensities are seen on T2W images (black arrows in Figure 3B and Figure 3C)
A 40-year-old male	Fever, headache, seizures, vertigo	Serum and CSF positive for dengue IgM, CSF lymphocytic pleocytosis, normal glucose, and slightly elevated proteins	Cortical areas of hyperintensities are observed in both parieto-occipital lobe on T2W (white arrows in Figure 4B) and FLAIR images (white arrows in Figure 4A and Figure 4C) of a 40-year-old male patient on day one of presentation to the hospital. New interval increase in hyperintense areas in DWI and T2W images, are identified in both parieto-occipital lobe and thalami of the 40-year-old male patient six days after presentation with dengue encephalitis (white arrows and black arrows, in Figure 5A and Figure 5B, respectively). Interval increased in the cortical hyperintensities are seen in both parieto-occipital lobe on DWI and FLAIR images (black arrows in Figure 6A and Figure 6B, respectively) of the 40-year-old male patient six days after presentation with dengue encephalitis
A 15-year-old male	Fever, headache, altered sensorium	Serum and CSF positive for dengue IgM, CSF lymphocytic pleocytosis, and normal glucose, slightly elevated proteins	Diffuse gyral areas of diffusion restriction are seen in both cerebral hemispheres on DWI (white arrows in Figure 7A and Figure 7B), predominantly in temporal lobe also involving both hippocampi on day one of presentation in a 15-year-old male patient with dengue encephalitis. Areas of diffusion restriction are also seen in both thalami on DWI with corresponding hyperintensities on T2W images (white short arrows in Figure 7A, Figure 7B, Figure 7C, respectively). MRI of a 15-year-old male patient on day six of admission. Increased gyral hyperintensities are observed in the posterior parietal lobe on T2W and DWI (white arrows in Figure 8A, Figure 8B, and Figure 8C)
A seven-year-old boy	Fever, increased sleepiness and difficulty to arouse, seizure	Serum and CSF positive for dengue IgM, CSF lymphocytic pleocytosis, and normal glucose	Within normal limits
A nine-year-old boy	Fever, headache, vomiting	Serum and CSF positive for dengue IgM, CSF lymphocytic pleocytosis, and normal glucose, slightly elevated proteins	Symmetrical hyperintensities are seen on FLAIR and T2W (white arrows in Figure 9A and Figure 9B, respectively) in both thalami in a nine-year-old male patient on day one of presentation with dengue encephalitis. Central areas of blooming are seen on SWI (Figure 9C). Diffusion restriction is also seen on DWI (white arrow in Figure 9D). Diffusion restriction is also seen on DWI images in the pons (white arrows in Figure 10A). Areas of blooming are seen on SWI (white arrow in Figure 10B). Hyperintense areas are seen in pons T2W sagittal images and FLAIR images (white arrow in Figure 10C and Figure 10D) in a nine-year-old male patient with dengue encephalitis

In case one, the 12-year-old female patient was back to normal in 21 days and could perform all daily life activities. Within a month, the 28-year-old patient was back to normal and capable of managing all daily chores on his own. The patient in case three, a 36-year-old woman, is still experiencing multifocal dystonia two months after being discharged. Unfortunately, the 17-year-old female patient passed away from her illness. The 40-year-old man recovered to his normal state in just 21 days and is now capable of managing all daily tasks on his own. The 15-year-old male patient is still suffering from short-term memory problems two months after being discharged. Otherwise, he is capable of managing everyday tasks on his own. In a month, the seven-year-old child was back to his routine and is now capable of managing his daily tasks on his own. Unfortunately, the nine-year-old boy died in a hospital.

## Discussion

Encephalitis is a rare but important neurological manifestation of dengue fever. Dengue fever can produce neurological impairment via numerous different pathways. First, the blood-brain barrier’s endothelial cells are impacted by immunological mediators released during active dengue infection. The barrier is breached by this impact, and the virus then directly enters the brain’s glial and neuronal cells. It may also enter the brain parenchyma via the bloodstream. Second, dengue encephalitis can also occur in the context of dengue shock syndrome and dengue hemorrhagic fever due to multisystemic dysfunction. Third, the dengue virus can cause encephalitis by ADEM, which is similar to a post-infectious immune response [5].

The diagnosis of dengue fever is more likely given the recent history of serum IgM ELISA-confirmed dengue infection (non-structural protein 1 antigen test or viral culture can also be done), fever, body pains, thrombocytopenia, and endemicity of the dengue virus in India. In an individual case, neurological signs and symptoms such as altered mental status, headaches, seizures, meningismus, nystagmus, focal neurological deficits, the presence of a Babinski’s sign, and vertigo are combined together with dengue symptoms to consolidate the whole picture of dengue viral encephalitis.

We reported eight cases of dengue encephalitis and described the MRI findings. In our case series of eight patients, we have shown the involvement of deep gray matter regions such as the basal ganglia and thalamus, periventricular white matter, subcortical gray matter, and cortical gray matter (supratentorium), and infratentorial regions such as brainstem (pons) and cerebellum. The dengue virus is also known to cause micro-hemorrhages inside the brainstem, cerebellum, and cerebral cortex. Many authors have studied the spectrum of MRI findings in dengue encephalitis. One study found that eight encephalitis patients had involvement of the cortex, brainstem, cerebellum, and ganglia-thalamic complex [6]. Weerasinghe and Medagam reported a case of dengue encephalitis with status epilepticus in 2019. They described subcortical white matter and cortical gray matter changes that were intense on T2W and FLAIR images [7]. Garg and colleagues reported the case of cortical laminar necrosis in a 12-year-old boy with a fever, impaired consciousness, and a dengue IgM antibody-positive CSF. Cortical laminar necrosis was gyriform enhancements. Lesions were present in the parieto-occipital regions and left frontal lobe. Gradient-echo sequences revealed bleeding in areas corresponding to gyriform enhancements a.k.a. cortical laminar necrosis. In addition, T2W hyperintensities were seen in both basal ganglia [8]. In 2017, Kumar et al. proposed naming the MRI findings of dengue encephalitis the “double donut sign,” as seen in a 23-year-old primigravida with a 10-day history of fever and three days of altered sensorium. On T1-weighted images, lesions in the bilateral thalami were hypointense. On T2W and FLAIR images, they were hyperintense. On SWI, the lesions showed intense diffusion restriction with bleeding [9]. The circumferential pattern of a hyperintense signal in the thalamus, which is one of the most common areas of affection seen in dengue encephalitis, led the author to propose naming the sign the “double donut sign” of dengue encephalitis. On DWI, Shah et al. (2018) also described a dengue double donut sign similar to our eighth case [10]. Many authors have found involvement of the thalamus, basal ganglia, brainstem, peri-ventricular white matter, and cortex on MRI in cases of dengue encephalitis, which are similar to our findings [11-13].

In our study of five male and three female patients, we found gray and white matter changes in the supratentorium, including deep gray matter (basal ganglia and thalamus), deep periventricular white matter, subcortical white matter, and sometimes cortical gray matter, as hyperintensities on T2W FLAIR images and hypo- (or iso-) intense lesions on T1-weighted images. Similarly, we observed the involvement of the infratentorial region, which includes the pons and white matter of the cerebellum, in one patient. In a subgroup of dengue encephalitis patients, MRI can be normal. In addition to the changes mentioned above, one of the eight patients had diffusion limitation on DWI and micro-hemorrhage shown as “blooming” on SWI. The 36-year-old woman still has multifocal dystonia two months after being discharged. Two months after being discharged, the 15-year-old has short-term memory loss. In the hospital, the 17-year-old girl and the nine-year-old child succumbed to the disease. The 28-year-old and seven-year-old patients were back to normal within a month. In 21 days, the 12-year-old child and 40-year-old man were back to normalcy.

## Conclusions

We discussed eight cases of dengue encephalitis along with the spectrum of MRI findings associated with each case. The clinical picture and CSF serological analysis help to rule out the differential diagnosis of herpes simplex encephalitis, scrub typhus, enteric fever, and JE. Our study found that MRI is an essential investigation in the management of dengue encephalitis. In rare severe cases, a cerebral herniation may occur due to cerebral edema. A baseline MRI should always be obtained in all cases suspected of dengue encephalitis and neurological features should be monitored to detect cerebral herniation timely. The management of the patient should be determined by the patient’s symptoms, and any neurological complaints in a person who has dengue fever should indicate that the person needs to be hospitalized immediately and given supportive care. Supportive treatment is the mainstay of care in dengue encephalitis. Dengue encephalitis can have a more favorable outcome for patients if they receive treatment as soon as possible.
